# Characterization of insulin crystalline form in isolated β-cell secretory granules

**DOI:** 10.1098/rsob.220322

**Published:** 2022-12-21

**Authors:** Seiya Asai, Jana Moravcová, Lenka Žáková, Irena Selicharová, Romana Hadravová, Andrzej Marek Brzozowski, Jiří Nováček, Jiří Jiráček

**Affiliations:** ^1^ Institute of Organic Chemistry and Biochemistry, Czech Academy of Sciences, Flemingovo nám. 2, 11610 Prague 6, Czech Republic; ^2^ Department of Biochemistry, Faculty of Science, Charles University, 12840 Prague 2, Czech Republic; ^3^ CEITEC, Cryo-Electron Microscopy and Tomography Core Facility, Masaryk University, Kamenice 5, 62500 Bohunice, Czech Republic; ^4^ York Structural Biology Laboratory, Department of Chemistry, University of York, Heslington, York YO10 5DD, UK

**Keywords:** crystallization *in vivo*, peptide hormone, subcellular vesicle, electron microscopy, secretory granules, insulin secretion

## Abstract

Insulin is stored *in vivo* inside the pancreatic β-cell insulin secretory granules. *In vitro* studies have led to an assumption that high insulin and Zn^2+^ concentrations inside the pancreatic β-cell insulin secretory granules should promote insulin crystalline state in the form of Zn^2+^-stabilized hexamers. Electron microscopic images of thin sections of the pancreatic β-cells often show a dense, regular pattern core, suggesting the presence of insulin crystals. However, the structural features of the storage forms of insulin in native preparations of secretory granules are unknown, because of their small size, fragile character and difficult handling. We isolated and investigated the secretory granules from MIN6 cells under near-native conditions, using cryo-electron microscopic (Cryo-EM) techniques. The analysis of these data from multiple *intra*-granular crystals revealed two different rhomboidal crystal lattices. The minor lattice has unit cell parameters (*a* ≃ *b* ≃ 84.0 Å, *c* ≃ 35.2 Å), similar to *in vitro* crystallized human 4Zn^2+^-insulin hexamer, whereas the largely prevalent unit cell has more than double *c*-axis (*a* ≃ *b* ≃ *c* ≃ 96.5 Å) that probably corresponds to two or three insulin hexamers in the asymmetric unit. Our experimental data show that insulin can be present in pancreatic *MIN*6 cell granules in a microcrystalline form, probably consisting of 4Zn^2+^-hexamers of this hormone.

## Graphical abstract

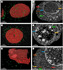

## 1. Introduction

Insulin is a small protein hormone that is essential for the uptake of blood glucose to muscle and adipose tissues. Insulin is produced in β-cells of the pancreatic Islets of Langerhans, synthesized firstly in endoplasmic reticulum as 110 amino acids preproinsulin. Subsequently, the preproinsulin's N-terminal signal sequence is cleaved and a single-chain 81 amino acid proinsulin is transported into the Golgi apparatus, where it is folded and stored in newly formed insulin storage granules [1]. It has been suggested that in Zn^2+^-rich secretory granules, single-chain proinsulin forms Zn^2+^-stabilized soluble hexamers [2]. Finally, specific convertases cleave out proinsulin's C-peptide to produce mature double-chain insulin that should still be present in the form of Zn^2+^-hexamer [3–6]. Insulin has low solubility at the mildly acidic pH that is in the secretory granules, which should accelerate the conversion from proinsulin and protect mature insulin from proteolysis. Moreover, it has been proposed that the high concentration of zinc ions and insulin in granules, and lower solubility of this hormone in this environment should promote aggregation, precipitation and crystallization of Zn^2+^-stabilized insulin hexamers stored in secretory granules *in vivo* [2,7–9].

The breakthrough in structural investigations of insulin was provided by Dorothy M. Crowfoot Hodgkin's group in 1969 [10]. They crystallized and solved the structure of the rhombohedral crystals of 2Zn pig insulin hexamer, paving the way for hundreds of *in vitro* structures of human insulin, its analogues and insulins from other species. It is known that different types of insulin hexamers, referred to as T_6_, T_3_R^f^_3_ and R_6_, depending on the conformation of insulin monomers forming the hexamer, can exist *in vitro* [6,11]. Insulin monomers can differ by the conformation of the B1–B6 residues of the B-chain. In the T conformation of insulin, residues B1–B6 are in an extended conformation, followed by a B7–B10 type II’ β-strand, followed by an invariant B9–B19 α-helix. In the presence of higher concentrations of small aromatic alcohols, such as phenol [12–14], the B1–B6 segment of insulin can adopt the α helical conformation, called the R state. At higher concentrations of SCN^−^, Cl^−^ or Zn^2+^ ions [15], or lower concentrations of cyclic alcohols [13,14,16], the long helix B in the R state shortens to the B3–B19 segment, and the B1–B3 residues ‘fray’ apart, giving what is called the R^f^ form of insulin. The specific type of insulin conformation in the hexamer can also be induced *in vitro* by the presence of neurotransmitters [17] or arginine [18], which are expected to be present in the granules [19–22]. Particular forms of the insulin hexamers *in vitro* give them characteristic thermodynamic stabilities [23,24]. Therefore, a specific type of crystalline insulin oligomer in the secretory granules may have an impact on the biodynamics of the insulin secretion process, and, ultimately, the bioavailability of the hormone *in vivo*.

Protein crystals in the living organism have always attracted the attention of the scientific community. The presence of crystalline materials *in vivo* inside cells, *in cellulo*, is rather a rare phenomenon, but has been known for several decades, with initial reports dating back to the nineteenth century when protein crystals in human tissue [25] and plant seeds [26] were first described. In general, aggregation or crystallization of proteins inside cells may have a detrimental effect on cells' viability and, hence, evolution favours soluble or membrane-anchored proteins [27]. Nevertheless, a fraction of proteins crystallizes in cells and their crystals can have functional consequences (see, for example, a review by Schönherr *et al*. [28]). However, only a few natively *in cellulo* crystallized proteins were detected in humans with, interestingly, all of them being linked to some pathological state. Accumulation of crystalline protein in the cytoplasm of histiocytes is associated with the crystal-storing histiocytosis [29]. Eye cataract can be caused by mutations in *γ*D crystallin that is prone to aggregation and crystallization in the lens [30]. Abnormal crystallization of mutated haemoglobin C in erythrocytes is known to cause haemolytic anaemia [31]. Charcot–Leyden crystals [25] are formed by galectin-10 crystallization in human tissues and are considered as a hallmark of inflammatory disorders [32]. Hexagonal Reinke crystals [33,34] are of unclear molecular nature. They are usually located within the cytoplasm of Leydig cells in the human testis and are considered for the diagnosis of tumours [35]. In a wide variety of neuromuscular diseases, crystalline inclusions of mitochondrial creatine kinase [36] represent a marked feature in the mitochondrial intermembrane space of skeletal muscle fibres.

Therefore, if insulin is indeed stored in a crystalline form in the β-cells of pancreatic Islets of Langerhans, it would represent a unique example of the functionally beneficial role of *in vivo* protein crystallization in the human body.

Regular objects reminiscent of microcrystals were already observed in pancreatic β-cell insulin secretory granules in the 1960s (e.g. [7] and [37] for a review). The first attempts to characterize crystalline insulin in secretory granules of the grass snake were made in the early 1970s by Lange *et al*. [38–40], based on the three-dimensional reconstruction from serial sections of the snake pancreatic tissue, and by optical diffraction. They found that snake pancreatic granules contain particles in the shape of rhombic dodecahedra. In 1978, Raška *et al*. [41] used similar electron microscopic techniques for an ultrastructural analysis of β-cell granules from the Langerhans Islets of the alligator. They identified four distinct morphological types of crystalline inclusions: rhombohedron, rhomb-dodecahedron, dipyramid and prism. While the first three types of crystals were attributed to insulin, prismatic crystals were similar to those of proinsulin crystallized *in vitro* [42]. These early studies provided a strong argument that insulin can be stored in the pancreas in a crystalline form. However, as the examined samples were prepared from tissues that were firstly fixed with glutaraldehyde and osmium tetroxide, dehydrated with alcohol and embedded in epoxy resin, it cannot be excluded that such harsh sample conditions affected these reported structural forms of insulin.

Indications that insulin may be present in the pancreas in a crystalline form prompted us firstly to study the quaternary structures of insulin in native secretory granules, using advances of X-ray synchrotron bright source. However, we were not able to register any X-ray diffraction from the isolated secretory granules from rat pancreatic Islets of Langerhans and from rat INS-1E cells. Nevertheless, X-ray fluorescence (XRF) scans revealed a striking difference in Zn^2+^ content between INS-1E secretory granules and native rat pancreatic Islet granules [43].

Herein, we expand the analyses of the content of isolated insulin secretory granules by the application of electron cryo-microscopic techniques that provide a unique opportunity for the structural analysis of a single insulin storage granule particle under near-native conditions. We have been able to characterize the crystal unit cells of crystalline insulin in native granule preparations and show that the shape and dimensions are in accordance with the size of some insulin hexamers determined from crystals of this hormone prepared *in vitro*.

## 2. Results and discussion

In the first stages of this study, we searched for the optimum material for the isolation of insulin secretory granules and for further structural analyses. The selection of the material was dictated by the need for the most native-like source of insulin secretory granules, but also for their most feasible and ethical source. Animal pancreatic tissue represents the most native-like source, but the isolation of a sufficient number of granules demands sacrificing dozens of animals, followed by challenging and expensive isolation methodology which requires disintegration of this tissue with large amounts of a collagenase [43]. Moreover, the final granule preparation from Islets of Langerhans is inevitably contaminated by secretory granules from other types of cells, e.g. glucagon-producing α-cells or somatostatin-producing δ-cells [44]. Therefore, we had to consider different permanent cultured β-cell lines, and their comparative evaluation, e.g. morphology and number of granules and ability to produce and secrete insulin, and, for further experiments, we selected the clonal BRIN-BD11 and INS-1E β-cell lines and insulinoma-derived MIN6 β-cells, all of them of rodent origin.

### 2.1. Comparison of permanent cell lines by focused ion beam/scanning electron microscopy microscopy

The comparison of insulin secretory granules in all studied permanent pancreatic cell lines was carried out using serial focused ion beam/scanning electron microscopy (FIB/SEM) microscopy, which allowed the automated acquisition of high-resolution three-dimensional data. Here, we were able to gradually image, and very efficiently reconstruct, the three-dimensional volume of the whole pancreatic β-cells, or part of their volume, and to evaluate the appearance of the granules and their content in the whole β-cell. Ultimately, this allowed us a comparison of different β-cell lines within the broader context of their morphology.

**Figure 1.**
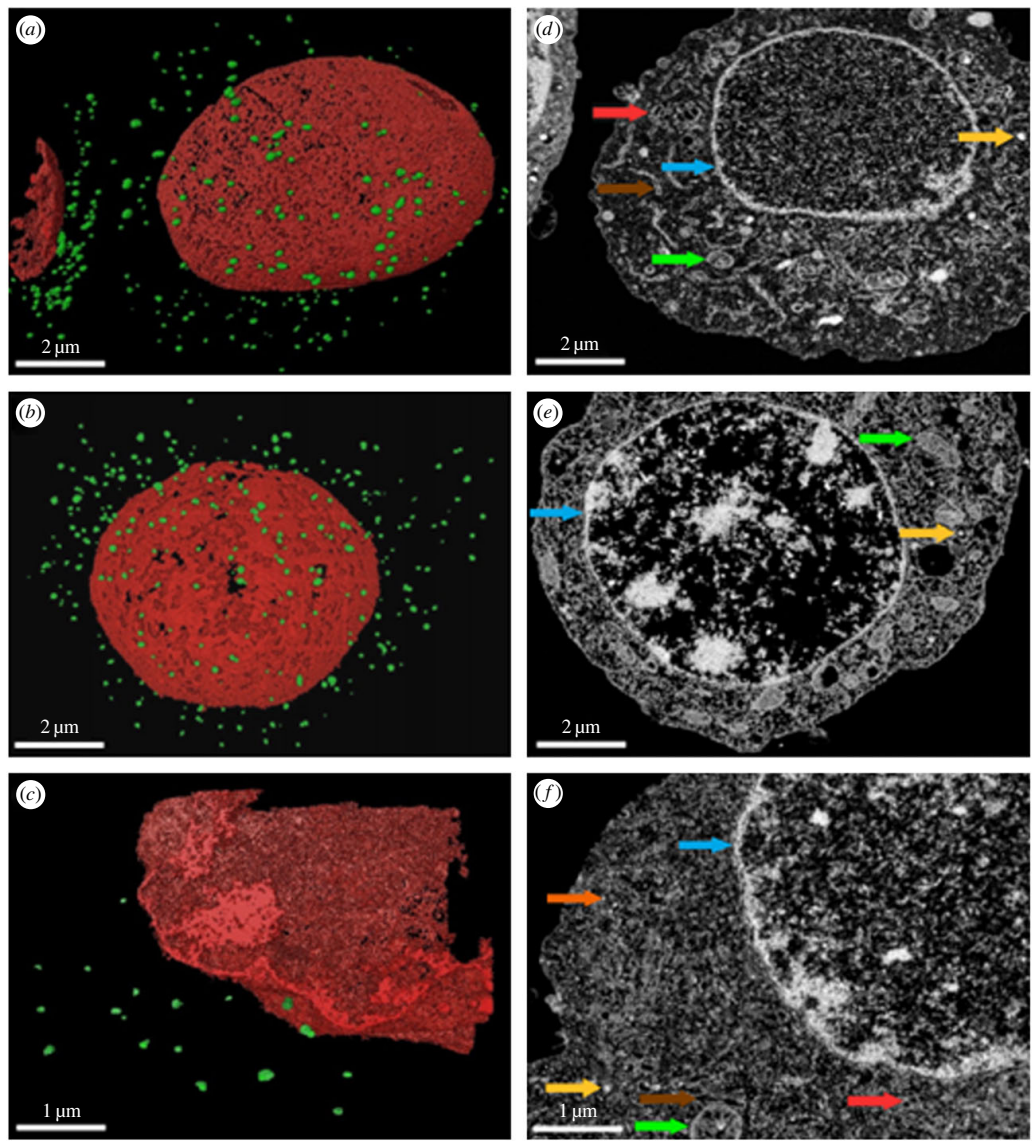
FIB/SEM microscopy images of the segmented examined β-cells. Images (*a*) (INS-1E), (*b*) (MIN6) and (*c*) (BRIN-BD11) show segmented cells where only nuclei (in red) and insulin secretory granules (in green) were visualized and segmented out from the context of the whole cell volume. Images (*d*) (INS-1E), (*e*) (MIN6) and (*f*) (BRIN-BD11) show scanning electron microscopy images of segmented cells contrasted by uranyl acetate and osmium tetroxide. Yellow arrows point at insulin secretory granules enveloped with a membrane vesicle. Blue arrows show nuclear envelope, green arrows point at mitochondria and red arrows show Golgi apparatus inside of the cell cytosol. Brown arrows point to the endoplasmic reticulum visible in INS-1E and BRIN-BD11 cells and the orange arrow points to a BRIN-BD11 cell ribosome.

**Figure 2.**
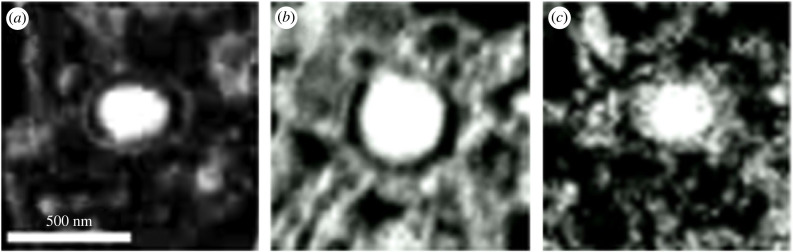
Magnified SEM images of typical secretory granules in the examined cell lines: (*a*) INS-1E, (*b*) MIN6 and (*c*) BRIN-BD11 cells.

We reconstructed and segmented whole pancreatic cells from the FIB/SEM data (electronic supplementary material, videos S1–S3), identified insulin secretory granules, and compared their populations among the studied cell lines in terms of number and size (figure 1). BRIN-BD11 cells clearly have a less dense population of insulin secretory granules, while INS-1E and MIN6 cells have a comparable number of granules per cell. We found that the apparent average size of granules (including the envelope) and insulin particles inside the granules is smaller in BRIN-BD11 (146 ± 22 nm and 94 ± 10 nm, respectively) than in INS-1E (238 ± 43 nm and 157 ± 33 nm) and MIN6 (250 ± 29 nm and 142 ± 12 nm) cells, which have a similar size of granules (figure 2).

In our other recent studies, we analysed the total insulin content in BRIN-BD11, INS-1E and MIN6 cells and in rat Langerhans Islets, and the insulin secretory properties of INS-1E and MIN6 cells after stimulation with glucose and different secretagogues [45]. This revealed that MIN6 cells are more glucose-responsive than INS-1E cells, both in terms of the quantity of secreted insulin and the reproducibility of results, despite a lower total insulin content in these cells. It is possible that the ability of INS-1E cells to secrete insulin in response to glucose is altered, possibly by an impaired proinsulin processing [43], and that higher intracellular concentration of (pro)insulin in INS-1E cells is caused by their reduced ability to secrete insulin. Therefore, considering all these results, and the initial analysis of the β-cells by FIB/SEM microscopy performed here, we selected the MIN6 cells for further isolation and analysis of insulin secretory granules, as they appear as a more native-like model of the β-cells than INS-1E or BRIN-BD11 cells.

### 2.2. Transmission electron microscopy (TEM) of insulin secretory granules from MIN6 cells

**Figure 3.**
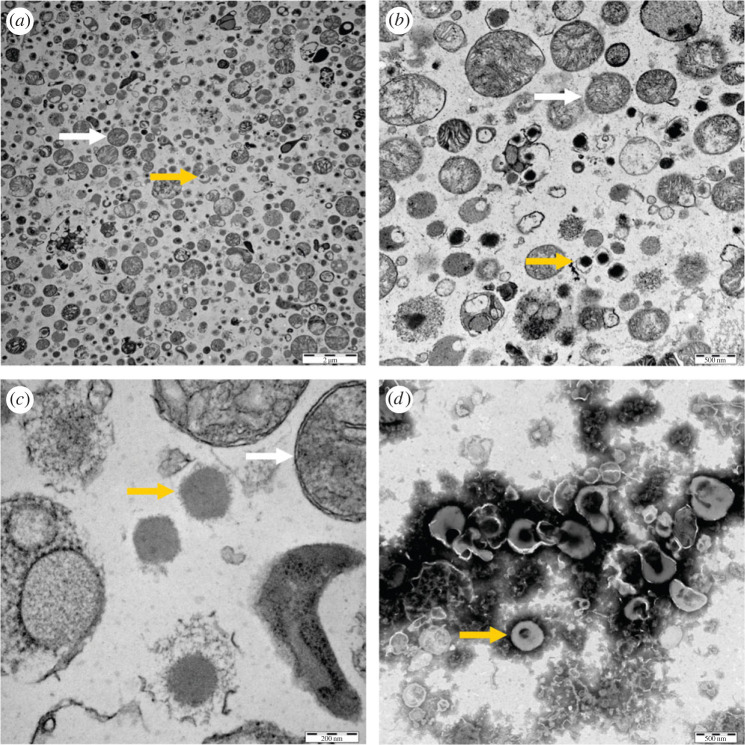
TEM images of isolated insulin secretory granule fraction from MIN6 cells. In (*a–c*), granule preparation is shown at three different magnifications (bars indicate 2 µm, 500 nm and 200 nm, respectively). Yellow arrows show different forms of granules (with or without the envelope), white arrows point to mitochondria. (*d*) An image of negative stained secretory granules having an intact envelope. Insulin particles are in dark objects inside of envelopes. The bar indicates 500 nm.

The insulin secretory granules from MIN-6 cells were isolated according to our protocol, described earlier by Dzianova *et al*. [43], and TEM images of isolated MIN6 cell secretory granules were collected (figure 3). Smaller particles representing insulin secretory granules were visible there (figure 3*a–c*), together with bigger mitochondria that were apparently copurified with insulin granules, a phenomenon already described by Brunner *et al*. [46]. They further optimized their purification protocol, extending it for an additional series of gradient centrifugations [47], as the presence of mitochondrial proteins would interfere with proteomics analyses of secretory granules. However, for our purpose here, the cryo-electron microscopy (Cryo-EM) and cryo-electron tomography (Cryo-ET) analyses of secretory granules, the presence of mitochondria in preparations did not represent any methodological problem. The TEM images showed (figure 3*b*) that some granules have an intact membrane, while other insulin-containing particles lack, completely or partially, their envelope, possibly due to damage during fractionation of the cells. It also seems that insulin particles without envelopes are still able to maintain their shape, even if some of them seem to be partly ‘dissolving’ (figure 3*c*).

### 2.3. Cryo-EM analyses of insulin secretory granules isolated from MIN6 cells

**Figure 4.**
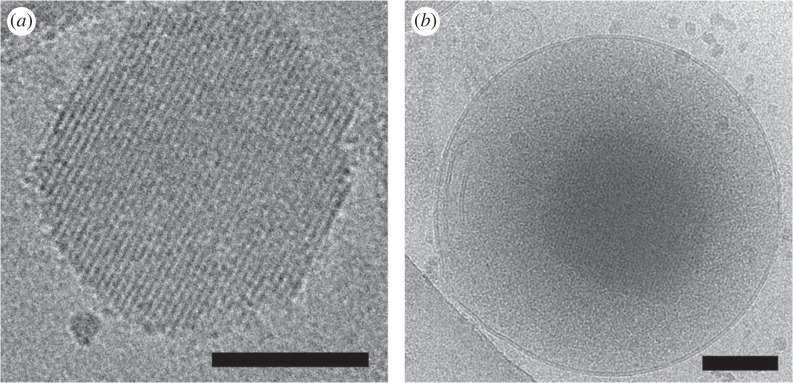
Cryo-EM images of the purified MIN6 cells insulin granules. Only the granule dense core is present in (*a*), whereas the whole granule is shown in (*b*). The bar corresponds to 100 nm in both insets.

The Cryo-EM imaging of the isolated granules showed dense globular objects with diameter 129–303 nm and average size of 198 ± 41 nm (based on analysis of 35 dense core particles), and with a clearly visible regular arrangement (figure 4). The crystalline cores observed by Cryo-EM are about 30% larger than the dimensions obtained from FIB/SEM data (see above). We attribute this difference to a significantly larger pixel size in the case of FIB/SEM experiments (3 nm with respect to 1.34 Å for Cryo-EM data) and thus lower precision of the distance measurement, and partly also due to the sample compression during embedding. The crystalline-like centres of the granules were either membrane-free (figure 4*a*) or encapsulated in the membrane (figure 4*b*). These differences (i.e. dense core only versus whole granule) are probably due to the osmotic or mechanical pressure during sample vitrification, as the residual traces of membrane in close proximity to the insulin-containing core were frequently observed.

Encouraged by the crystalline-like character of the granular core, we attempted the collection of the continuous rotation electron diffraction tomography (EDT) data, to determine the insulin structure in this crystalline lattice. However, this experiment was hampered by the small size of the crystalline cores. More importantly, we did not observe reflection peaks beyond approximately 5 Å, even in the static images. Therefore, we subsequently focused on the analysis of the low-resolution diffraction to determine the unit cell parameters of these crystalline cores. Here, we either collected discontinuous EDT data (electronic supplementary material, video S4) within ±40° tilt range (with 2° step) and image tracking step after every tilt. In addition, we also collected Cryo-ET data (electronic supplementary material, video S5) within the same tilt range as the EDT data, aligned the tilt series, and calculated the power spectra from the region containing the dense core. The data analysis relied only on the directions of the reflections, not on the precise determination of their intensities. In total, we analysed 20 granule dense cores and found that, in 19 cases, the crystals form a rhombohedral lattice with unit cell dimensions *a* ≃ *b* ≃ *c* ≃ 96.5 Å, *α* ≃ 90°, *β* ≃ *γ* ≃ 120°, whereas, in the last case, the parameters of the rhombohedral lattice were *a* ≃ *b* ≃ 84.0 Å, *c* ≃ 35.2 Å, *α* ≃ *β* ≃ 90°, *γ* ≃ 120°. The latter, smaller lattice is in good agreement with the lattice parameters of human 4 Zn^2+^-insulin hexamers (*a* = *b* = 80.953 Å, *c* = 37.636 Å) crystallized *in vitro*, analysed by X-ray diffraction and reported firstly in T_3_R^f^_3_ form by Smith *et al*. [48], with one insulin hexamer in the asymmetric unit (AU) (space group *R*3).

However, the dimension of the *c*-axis is more than double for most of the crystals analysed here, which suggests that *in cellulo* larger packing assemblies of insulin hexamers are preferred over ‘individual’ hexamers. Assuming the *R*3 space group for this larger lattice, the Matthews coefficients which depict protein crystal Å^3^/Dalton parameter, typically approximately 2.4 Å^3^/Dalton for 47% crystal solvent content [49], would be 3.8 Å^3^/Dalton (approx. 68% solvent) for two hexamers in the crystal AU, and 2.5 Å^3^/Dalton—corresponding to approximately 51% solvent in the crystals for three hexamers/AU. If higher *R*32 trigonal symmetry is considered, Matthews coefficients' values would be 3.8 Å^3^/Dalton (approx. 68% solvent) for one hexamer/AU and 1.9 Å^3^/Dalton (approx. 35% solvent) for two hexamers/AU.

Given the size of the larger unit cell, insulin arrangement composed e.g. of two (or more) hexamers on top of each other as seen, for example, in the *in vitro* crystal structure by Murayoshi *et al*. (PDB: 3W80, *a* = *b* = 82.7 Å, *c* = 68.1 Å, *α* ≃ *β* ≃ 90°, *γ* ≃ 120°) would explain the experimental data. Indeed, evidence of ‘dodecameric-like’ alligator insulin in microcrystals *in cellulo* was already provided earlier by Raška *et al*. [41]. In the insulin crystal structure by Murayoshi *et al*., the crystal lattice is formed by staggered columns of tightly packed (on top of each other) hexamers. Therefore, it can be envisaged here that a similar, tight two-hexamer basic packing unit, leading to a torus-like repeated hexamer column arrangement, can occur in the granules' lattice. This would ensure an additional level of stability, superior to a single insulin hexamer (or dimer) crystal packing motif, also prolonging the storage lifetime of insulin *in vivo.* This would be consistent with our findings that the crystalline insulin structure in isolated granules remained compact, even after loss of the granular envelope (figure 3). However, some other crystal packing arrangements involving, for example, three hexamers, cannot be excluded.

It is also tempting to consider here the T_3_R^f^_3_ form of insulin storage hexamer that was found *in vitro* not only in the 4Zn^2+^ insulin oligomer [48], but also in insulin hexamers obtained in the presence of neurotransmitter serotonin [17] and proinsulin post-processing by-product arginine [18]. Both these T_3_R^f^_3_ hexamer-stabilizing ligands are expected to be common components of insulin storage granules [19–22]. However, the tight column-like packing of insulin hexamers in the Murayoshi *et al*. crystal structure (PDB: 3W80) has been allowed by the compact T_6_ form of these hexamers. Therefore, the expansion of this unit cell from *in vitro* approximately 68.1/82.7 Å to approximately 96.5 Å that is observed in the dominant granules’ crystal lattice found in this study, may result from a need to accommodate possible, and relatively bulkier, T_3_R^f^_3_-like insulin hexamers.

Small amino acids’ sequence differences between mouse/rat insulin studied here and the human hormone (electronic supplementary material, figure S1) should not prevent the occurrence of similar tertiary and quaternary structures of both hormones.

The estimated unit cell parameters and the crystalline core size volume determined by Cryo-ET allow us to estimate that a single MIN6 cell granule can store approximately 60 000 insulin molecules, which is not too far from the 200 000 human insulin molecules per cell estimated much earlier in 1984 [50].

### 2.4. Cryo-FIBM and Cryo-ET analyses of cross-sections from MIN6 cells

**Figure 5.**
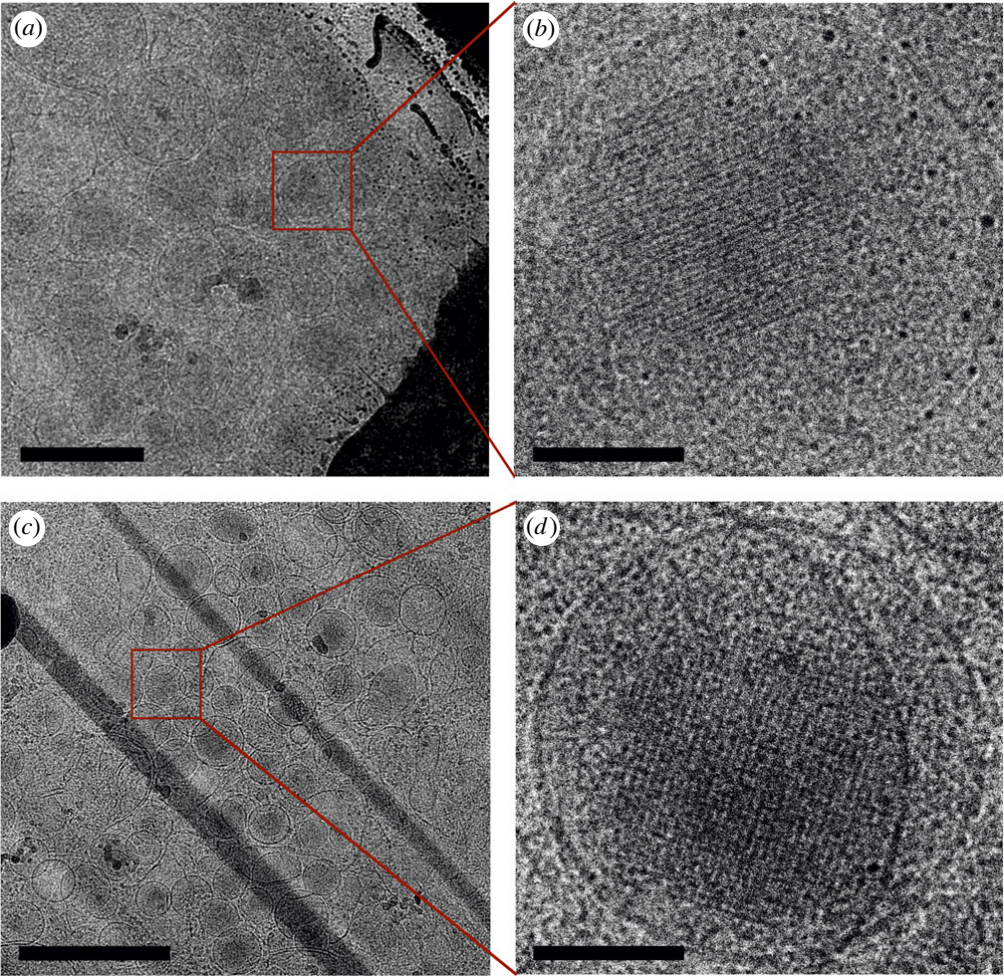
Crystalline core visualized *in cellulo* within insulin secretory granules of MIN6 cells. Cellular lamellae prepared by cryo-FIB depicting thin sections of MIN6 cells (*a*,*c*). Images from the cryo-ET tilt series showing a detailed view on the granule crystalline core alongside the zonal planes of the insulin crystals (*b*,*d*). Scale bars corresponds to 1 µm in (*a*) and (*c*) and 100 nm in (*b*) and (*d*).

Finally, we used cryo-focused ion beam micromachining (Cryo-FIBM) and Cryo-ET to visualize the granules in the cross-section of the MIN6 cell under near-native conditions. Similarly to the imaging of purified granules, we searched for the granule orientation where the high-symmetry axis was parallel to the beam direction. We found that the granular dense core in whole MIN6 cells indeed has a regular arrangement (figure 5), similar to isolated granules.

## 3. Conclusion

We isolated insulin secretory granules from mouse β-cell-derived MIN6 cells under close-to-native conditions, to assure the minimum effect of the experimental conditions on the storage form of insulin in the granules. Employing Cryo-EM, we detected a three-dimensional regular arrangement in the dense core particles within the secretory granules, indicating the presence of crystalline material. We were able to identify two types of rhombohedral lattices in these microcrystals: a minor lattice with the unit cell dimensions *a* ≃ *b* ≃ 84.0 Å, *c* ≃ 35.2 Å, *α* ≃ *β* ≃ 90°, *γ* ≃ 120°, and a highly predominant lattice form with the unit cell dimensions *a* ≃ *b* ≃ *c* ≃ 96.5 Å, *α* ≃ 90°, *β* ≃ *γ* ≃ 120°. The minor form of the lattice corresponds relatively well to the presence of the hexameric 4Zn^2+^ insulin crystal form, hence possibly to the T_3_R^f^_3_ hexamer of this hormone, while the major lattice form, with almost twice the length of the *c-*axis, indicates the presence of higher oligomeric packing of insulin forms, for example two toroidal hexamers on top of each other (pseudo ‘dodecamer’). Our findings clearly confirm previous assumptions that stored insulin is present in the cell in crystalline form, also providing unambiguous details on the *in cellulo* crystal lattice parameters, and its hexamer packing, characterized in the most native experimental conditions to date. Considering the current rapid developments in cell biology and cryo-microscopic imaging techniques, our findings could initiate new studies investigating, for example, the relationship between the type of insulin structural forms (e.g. crystalline or amorphous) in secretory granules and the ability of β-cells to secrete insulin.

## 4. Methods

### 4.1. Cell cultures and cell cultivation

The clonal β-cell line BRIN-BD11 (Sigma-Aldrich, Cat no. 10033003) is a hybrid line formed by electrofusion of a primary culture of rat pancreatic β-cells (New England Deaconess Hospital, NEDH) with RINm5F cells [51,52]. The cells were cultured in a humidified atmosphere containing 5% CO_2_ and at 37°C in a complete medium, composed of RPMI 1640 medium supplemented with 10% heat-inactivated fetal bovine serum (FBS), 2 mM l-glutamine, 10 mM HEPES, 100 U ml^−1^ penicillin and 100 U ml^−1^ streptomycin.

The rat clonal β-cell line INS-1E [53] (AddexBio, Cat no. C0018009) derived from parental INS-1 cells [54] was cultured in a humidified atmosphere containing 5% CO_2_ and at 37°C in a complete medium, composed of RPMI 1640 medium supplemented with 10% heat-inactivated FBS, 1 mM sodium pyruvate, 50 mM 2-mercaptoethanol, 2 mM l-glutamine, 10 mM HEPES, 100 U ml^−1^ penicillin and 100 U ml^−1^ streptomycin.

Mouse insulinoma-derived MIN6 cells were a kind gift from the Miyazaki laboratory, Osaka University, Japan [55] and were cultivated in a complete medium, composed of DMEM high glucose medium, supplemented with 10% heat-inactivated FBS, 50 mM 2-mercaptoethanol, 2 mM l-glutamine, 100 U ml^−1^ penicillin and 100 U ml^−1^ streptomycin.

### 4.2. Cell fixation and resin embedding

Permanent pancreatic cells were cultivated until 80% confluency, collected, and pelleted by spinning at 500 g at 37°C. Each cell pellet was fixed with 2.5% glutaraldehyde in 0.1 M phosphate buffer saline (PBS), washed and mixed with 2% agar. The cell pellet in agar was fixed with 2% OsO_4_ in 0.1 M PBS, washed and soaked with 1% uranyl acetate. Stained cells were gradually dehydrated in 40–100% ethanol and transferred into propylenoxide. Both procedures were performed on ice. Propylenoxide was gradually exchanged with durcupan with constant shaking and the samples were allowed to polymerize 48 h at 60°C.

### 4.3. Resin sample processing and preparation for focused ion beam/scanning electron microscopy

Resin blocks with a cellular sample were roughly cut out by a razor blade and fixed on the top of the electron microscope stub. Resin cellular blocks were trimmed, and the block face gently smoothed with a glass knife on ultramicrotome. The smoothened cellular blocks (10 nm) were sputtered with 10 nm of inorganic gold.

### 4.4. Serial section electron microscopy

The Helios 4 DualBeam FIB–SEM microscope (Thermo Fisher Scientific), equipped with backscattered electron in-column detector (ICD) and Auto Slice and View software (Thermo Fisher Scientific), was used for collection of raw serial section electron microscopy (SSEM) data by a method of the alternation of thin specimen slice milling by focused gallium ion beam and high-resolution imaging of specimen face by electron beam.

The area of interest was covered with a 1–2 mm of organic platinum protective layer, created by the gas injection system (GIS). A fiducial marker for tracking and image alignment was created by milling of the cross-shape pattern into a 1–2 mm GIS rectangle protective layer.

The microscope stage was tilted to 52° to get the specimen face perpendicular to the ion beam source. The specimen block with dimensions: *x* = 20–30 µm, *y* = 20–30 µm and *z* = 10–20 µm was revealed by trenches' creation around three sides of the block (front, left and right). The trenches were created by FIB at 7 nA current. The block face for SEM imaging was smoothed by FIB current set in the range of 1.2–2.4 nA.

The following settings for SSEM imaging were defined: magnification ranged between 7K-fold and 9K-fold, image resolution was 3072 × 2048, dwell time was 6 ms, image pixel size ranged between 3 × 3 nm and 9 × 9 nm. Backscattered electrons were detected by ICD.

The following settings for FIB milling were defined: single slice thickness ranged from 10 to 20 nm, FIB current was set to 0.75 nA.

### 4.5. Data processing and segmentation of insulin granules in slices from cells

SSEM data collected in TIFF image format were semi-automatically aligned; the region of interest in *xyz* dimensions was extracted out of the dataset and the pancreatic cell nuclear envelope and visible insulin granules were segmented out and visualized in Amira software (Thermo Fisher Scientific). Next, on a sample of 30 secretory mature granules (with the insulin content separated from the envelope by a visible ‘halo’) in each of the cell types, BRIN-BD11, INS-1E and MIN6, we measured the average size (± s.d.) of the granules (including their envelope) and then the average size (± s.d.) of the insulin particles inside the granules. An example of the measurements in INS-1Es is shown in electronic supplementary material, figure S2.

### 4.6. Isolation of secretory granules from MIN6 cells

The isolation of granules was performed according to the protocol published by Brunner *et al*. [46] and described in detail in Dzianova *et al*. [43]. The final pellet of isolated insulin secretory granules was re-suspended in buffer composed of 0.27 M sucrose, 10 mM MOPS/1 M Tris, pH 6.8 (SMT buffer), kept at 0–4°C without freezing, and used for further analyses within the next 24 h.

The protein content in the granule fraction was determined by the Bradford assay. The proteins from isolated and lysed granules were separated on sodium dodecyl sulphate–polyacrylamide gel electrophoresis (SDS–PAGE) (12% gels) and checked by using the standard Western blotting procedure for insulin (anti-insulin antibody, L6B10, Cell Signalling) and betagranin (anti-chromogranin A antibody directed against N-terminal amino acids, ab45179, Abcam) content, according to Dzianova *et al*. [43] (electronic supplementary material, figure S3).

### 4.7. Chemical fixation and transmission electron microscopy of isolated insulin secretory granules from MIN6 cells

The fixation and examination of isolated granules were carried out as previously described in Dzianova *et al*. [43]. Briefly, the suspension of freshly isolated granules in SMT buffer was fixed with 2.5% glutaraldehyde in 0.1 M cacodylate buffer (pH 7.5) at room temperature (RT) for 15 min. Then the granule suspension was centrifuged at 120 g for 4 min. A new batch of 2.5% glutaraldehyde in 0.1 M cacodylate buffer was added and then the pellets were post-fixed in 1% osmium tetroxide, dehydrated in ethanol and embedded in Agar 100 epoxy resin. Ultrathin sections (70 nm) were cut with a diamond knife on a Leica UC6 ultramicrotome (Leica Microsystems, Wetzlar, Germany). The ultrathin sections were collected on parlodion-coated microscopy grids and contrasted, using saturated uranyl acetate and lead citrate. The isolated granules were also negative stained with 2% sodium phosphotungstate on parlodion-carbon-coated grids. The samples were analysed with the transmission electron microscope JEOL JEM-1011 device at 80 kV beam acceleration voltage.

### 4.8. Preparation of insulin secretory granules isolated from MIN6 cells for Cryo-EM

The suspension of freshly isolated MIN6 granules in SMT buffer (kept strictly at 0–4°C) was applied to the freshly plasma-cleaned holey carbon TEM grids (Quantifoil, Cu, 200 mesh, R2/1) and vitrified by plunge freezing into liquid ethane, using Vitrobot IV (ThermoScientific). The sample was incubated for 30 s on the grid in the instrument climate chamber (4°C, 100% rel. humidity) with prior blotting from both sides with filter paper. The grids with vitrified specimens were transferred to liquid nitrogen, mounted into the Autogrid cartridge and loaded into the transmission electron microscope for imaging.

### 4.9. Cryo-focused ion beam preparation of lamellas from MIN6 cells for Cryo-TM

Freshly plasma-cleaned holey carbon TEM grids (Quantifoil, Au, 200 mesh, R2/2) were placed into 10 mm cultivation wells (ThermoScientific) and supplemented with 100 ml of cell suspension, containing approximately 10 000 MIN6 cells. The wells were placed into the incubator (37°C, 5% CO_2_) for 16 h to allow adhesion of MIN6 cell to the grid surface. Subsequently, the grids were transferred to Vitrobot IV, blotted against non-absorptive blotting material, and vitrified into liquid ethane. The grids were mounted into the AutoGrid cartridge and loaded into the Versa 3D FIB/SEM microscope (ThermoScientific) for lamella preparation. Cells positioned close to the centre of the grid square were selected for lamella preparation by FIB. The sample surface was coated with a protective layer of metallo-organic platinum (GIS) and sputter coated with iridium. The lamella was prepared by gradual milling from both sides, with gradual decrease of the distance between the milling patterns and the milling current. The final polishing step was carried out with 10 pA milling current and was used to refine the lamella to 150–250 nm thickness. The grid was then transferred to the transmission electron microscope for Cryo-ET.

### 4.10. Cryo-TEM imaging

The cryo-transmission electron microscopy (Cryo-TEM) data were collected, using either Talos Arctica (operated at 200 kV) or Titan Krios (operated at 300 kV) transmission electron microscopes (ThermoScientific). The microscopes were equipped with post-column energy filter (Gatan) and K2, or K3, respectively, (Gatan) direct electron detector cameras. Single Cryo-TEM images of purified granules were collected with the overall dose less than 15 e/A^2^. The Cryo-ET and discontinuous EDT data were collected using SerialEM software. The acquisition of the tilt series was started at −40° stage tilt and the stage were incrementally rotated by 2° until +40° stage tilt. The overall dose per tilt series did not exceed 15 e/A^2^. Cryo-ET data were collected with defocus of −0.5 µm or −2.5 µm, respectively. The acquired frames were first corrected for the drift and beam-induced motion MotionCor2 (PMID: 28250466); contrast transfer function parameters were estimated using the GCTF program (PMID: 26592709), and the tilt series alignment and tomogram reconstruction were carried out in Imod. The granule dense core was cropped from the aligned tilt series collected with −0.5 µm, Fourier transformed, and the resulting power spectra were converted to SMV file format, using the in-house Python script for further processing in X-ray detection software (XDS). EDT data were collected on the K3 camera, operated in counting mode as single images per individual tilt angles. The resulting tilt series was first rotated in imod, to align the tilt axis with the image *y-*axis and cropped to square images. Subsequent data analysis to determine the unit cell parameters was carried out in XDS.

## Data Availability

The data are provided in electronic supplementary material [56].
